# *Sphingomonas abietis* sp. nov., an Endophytic Bacterium Isolated from Korean Fir

**DOI:** 10.4014/jmb.2303.03017

**Published:** 2023-07-18

**Authors:** Lingmin Jiang, Hanna Choe, Yuxin Peng, Doeun Jeon, Donghyun Cho, Yue Jiang, Ju Huck Lee, Cha Young Kim, Jiyoung Lee

**Affiliations:** 1 Korean Collection for Type Cultures (KCTC), Biological Resource Center, Korea Research Institute of Bioscience and Biotechnology, Jeongeup 56212, Republic of Korea; 2 Department of Biosystem and Bioengineering, KRIBB School of Biotechnology, University of Science and Technology (UST), Daejeon 34113, Republic of Korea; 3 Present address: National Key Laboratory of Plant Molecular Genetics, CAS Center for Excellence in Molecular Plant Sciences, Chinese Academy of Sciences, Shanghai 200032, P.R. China

**Keywords:** Polyphasic taxonomy, Korean fir, phylogeny, AntiSMASH, endophytic bacterium

## Abstract

PAMB 00755^T^, a bacterial strain, was isolated from Korean fir leaves. The strain exhibits yellow colonies and consists of Gram-negative, non-motile, short rods or ovoid-shaped cells. It displays optimal growth conditions at 20°C, 0% NaCl, and pH 6.0. Results of 16S rRNA gene-based phylogenetic analyses showed that strain PAMB 00755^T^ was most closely related to *Sphingomonas chungangi* MAH-6^T^ (97.7%) and *Sphingomonas polyaromaticivorans* B2-7^T^ (97.4%), and ≤96.5% sequence similarity to other members of the genus *Sphingomonas*. The values of average nucleotide identity (79.9–81.3%), average amino acid identity (73.3–75.9%), and digital DNA–DNA hybridization (73.3–75.9%) were significantly lower than the threshold values for species boundaries; these overall genome-related indexes (OGRI) analyses indicated that the strain represents a novel species. Genomic analysis revealed that the strain has a 4.4-Mbp genome encoding 4,083 functional genes, while the DNA G+C content of the whole genome is 66.1%. The genome of strain PAMB 00755^T^ showed a putative carotenoid biosynthetic cluster responsible for its antioxidant activity. The respiratory quinone was identified as ubiquinone 10 (Q-10), while the major fatty acids in the profile were identified as C_18:1_*ω*7*c* and/or C_18:1_*ω*6*c* (summed feature 8). The major polar lipids of strain PAMB 00755^T^ were diphosphatidylglycerol, phosphatidylethanolamine, sphingoglycolipid, and phosphatidylcholine. Based on a comprehensive analysis of genomic, phenotypic, and chemotaxonomic characteristics, we proposed the name *Sphingomonas abietis* sp. nov. for this novel species, with PAMB 00755^T^ as the type strain (= KCTC 92781^T^ = GDMCC 1.3779^T^).

## Introduction

*Sphingomonas* is a type of Gram-negative, rod-shaped or ovoid bacteria in the family *Sphingomonadaceae* and order *Sphingomonadale*. The genus was first introduced by Yabuuchi in 1990 [1] with *Sphingomonadaceae paucimobilis* as the type species (basonym: *Pseudomonas paucimobilis*). In 2001, further classification based on phylogenetic and chemotaxonomic analyses subdivided the genus *Sphingomonas* into three new genera: *Sphingobium*, *Novosphingobium*, and *Sphingopyxis* [2]. As of February 2023, there were 152 published species in the genus *Sphingomonas*, characterized by the presence of Q-10 as the predominant isoprenoid and C_16:0_, C_14:0_-2OH, and C_18:1_ω7c and/or C_18:1_ω6c (summed feature 8) as the major fatty acids. *Sphingomonas* species have been found in various environmental sources, including water, soil [3-5], sand [6], fish ponds [7], fruit [8], plants [8-10], hair [11], and air [12, 13]. Some species in this genus have been identified as radiation resistant [14], able to survive in procymidone-contaminated environments [15], and able to thrive in oil-contaminated soil [16], lead–zinc mines [17], and bark [18]. Additionally, some species were found to have agar-degrading [19], chitin-degrading [20], and polycyclic aromatic hydrocarbon-degrading [21] activities.

In this study, we isolated a new bacterial strain designated PAMB 00755^T^ from the leaves of Korean fir (*Abies koreana*) growing on Hallasan Mountain, Jeju Island, South Korea. In 2011, the International Union for Conservation of Nature (IUCN) identified a Korean fir (*Abies koreana*) as an endangered species in Korea. In recent years, to investigate the bacterial community associated with Korean fir and identify potential bioactive secondary metabolites of the host, we collected leaves from both healthy and diseased firs and analyzed the endophytic bacterial community [10, 22, 23]. In this study, through a polyphasic taxonomic analysis of leaves from a healthy fir, we characterized strain PAMB 00755^T^ as a novel species in the genus *Sphingomonas*.

## Materials and Methods

### Isolation and Ecology

Leaves were collected from the Korean fir growing on Hallasan Mountain (33°21'42''N, 126°31'45''E) on Jeju Island, South Korea. To prepare the sample, five grams of leaves were subjected to surface sterilization using 1%NaOCl solution. The leaves were rinsed five times with distilled water. The sterilized leaves were then homogenized using a blender with 10 ml of 1× phosphate-buffered saline (PBS). To remove any debris, the resulting mixture was filtered through four layers of sterile cheesecloth and then serially diluted using a standard dilution method with 1× PBS buffer. A volume of 100 μl from the sample solution was evenly spread onto Reasoner's 2A agar (R2A, Difco) plates, followed by incubation at 25°C for 1 week. Single colonies were then re-streaked onto fresh R2A medium. Strain PAMB 00755^T^, which formed circular, smooth, yellow colonies, was selected for the following study. The strain was stored at -80°C in 10% skimmed milk. It is currently accessible through the Korean Collection for Type Cultures (KCTC 92781^T^) and the Guangdong Microbial Culture Collection Center (GDMCC 1.3779^T^). Unless otherwise noted, bacterial cells were cultured on R2A for 3–4 days before performing subsequent tests.

### 16S rRNA Gene Sequence Analysis

The extraction of genomic DNA from the bacteria was performed to serve as a template for PCR amplification of the 16S rRNA gene. Universal bacterial primers, specifically 27F and 1492R, were used for the amplification process. The PCR products were sequenced by a commercial company (Macrogen Inc., Korea) using the primers 27F, 518F, 800R, and 1492R [24]. The almost full length of the 16S rRNA sequence was assembled using Vector NTI software (1.6.1). The obtained sequences were compared in the EzBioCloud and the NCBI database [25]. The alignment of multiple sequences was performed using BioEdit software. Subsequently, the construction of the phylogenetic tree was carried out using the Molecular Evolutionary Genetics Analysis (MEGA) software version 11.0. The neighbor-joining (NJ), maximum likelihood (ML), and minimum parsimony (MP) methods were used for this analysis, with 1,000 bootstrap iterations [26]. The outgroup was *Usitatibacter rugosus* 0125-3^T^.

### Whole-Genome Characteristics

Genomic DNA was extracted as previously described [10]. For whole-genome sequencing, the PacBio RSII system (Pacific Biosciences, Inc., USA) as well as an Illumina sequencing platform by Macrogen, Inc. (Korea), were utilized. The obtained raw data were *de novo* assembled using SMRT LINK (12.0) software. To evaluate the quality, completeness, and contamination of the assembled genome, the Microbial Genomes Atlas (MiGA) webserver was employed [27]. The NCBI’s Prokaryotic Genome Annotation Pipeline (PGAP) was utilized for genome annotation. To determine the overall genomic relatedness values between strain PAMB 00755^T^ and closely related strains, various tools were employed. The Genome-to-Genome Distance Calculation (GGDC) webserver (http://ggdc.dsmz.de/distcalc2.php) was used for digital DNA–DNA hybridization (dDDH) [28]. The AAI calculator (http://enve-omics.ce.gatech.edu/aai/) was used for average amino acid identity (AAI) [29], and the ANI calculator (http://enve-omics.ce.gatech.edu/ani/) was used for average nucleotide identity (ANI). Based on the latest bacterial core gene set and pipeline (UBCG) analysis, a comprehensive phylogenomic tree was constructed. This analysis incorporated 92 core genes, ensuring a robust representation of the evolutionary relationships among the bacterial strains [30]. The outgroup was *Usitatibacter rugosus* 0125-3^T^.

### Chemotaxonomic Characterization

Chemotaxonomic features, including cellular fatty acids, polyamines, isoprenoid quinones, and polar lipids, were investigated. For cellular fatty acid analysis, approximately 40 mg of fresh cells from the third quadrant of the streaked plates were collected. The cellular fatty acids were extracted as previously described [22] using a MIDI Sherlock Microbial Identification System (6.0). The samples were subjected to gas chromatography using a 6890N gas chromatography system (Agilent Technologies, USA), and the data were identified using the TSBA6 database of the Microbial Identification software package [31]. Polyamines were extracted from 0.1 g of freeze-dried cells and analyzed by comparison to spermine, putrescine, spermidine, and homospermidine standards in Ace Emzyme Inc. (Korea). Isoprenoid quinones were extracted using the method outlined by Collins *et al*. (1980) [32] and subsequently analyzed by reverse-phase high-performance liquid chromatography. The extraction of polar lipids was carried out from 0.1 g of freeze-dried cells using a chloroform–methanol (2:1). Following the extraction, two-dimensional thin-layer chromatography was performed to separate the polar lipids that were obtained. To visualize the polar lipids on the TLC plates, four different spray reagents were used: molybdenum blue (Sigma-Aldrich, USA), 4% phosphomolybdic acid reagent, 0.2% ninhydrin (Sigma-Aldrich), and Dragendorff ’s solution.

### Phenotypic Characteristics

After culturing strain PAMB 00755^T^ on R2A medium for 3 days, the morphological features of the strain were examined using scanning electron microscopy (SEM) at the Korean Basic Science Institute in Chuncheon. To examine motility, a semi-solid R2A medium supplemented with 0.4% agar was used. The Gram staining procedure was conducted using a Gram staining kit (Difco), following the manufacturer’s instructions. Catalase activity was assessed by observing bubbles production upon addition of a 3% (v/v) hydrogen peroxide solution to fresh cells. Oxidase activity was determined by observing the development of a purple color using an oxidase reagent kit. The strain was grown on various media, comprising R2A, nutrient agar (NA), potato dextrose agar (PDA), trypticase soy agar (TSA), marine agar 2216 (MA), and Luria-Bertani (LB) agar to determine the optimum medium. The strain was grown on PDA at 4, 10, 15, 20, 25, 30, 37, 40, 45, 50 and 60°C for 7 days to determine the optimal temperature. Salt tolerance was tested by growing the strain in R2A broth with concentrations from 0 to 15% (w/v) in increments of 1% [33], and pH tolerance was tested by growing the strain at a pH from 3.0 to 12.0 in increments of 1. Other biochemical features were tested using API 20NE (bioMérieux; substrate utilization, France) or API ZYM (bioMérieux; enzyme activities; NaCl 0.85% medium) and API 50CH (bioMérieux; production from carbohydrates) following the manufacturer’s instructions.

## Results and Discussion

### Phenotypic, Physiological, and Biochemical Characteristics

Strain PAMB 00755^T^ exhibited optimum (and robust) growth on both PDA and R2A media, limited growth on NA, and no growth on TSA, LB, or MA. The cells are Gram-negative, non-motile, and short rods or ovoid (0.3–1.0 × 0.4–1.2 μm) without flagella (Fig. S1). Colonies were yellowish and ranged from 2–4 mm in diameter after being grown for 3 days on PDA medium. The strain grew at pH 4.0–9.0 (optimum pH, 6.0), 0–1% of NaCl (optimum NaCl concentration, 0%), and 4–25°C (optimum temperature, 20°C). It tested positive for catalase and oxidase activity. Other characteristics that distinguished strain PAMB 00755^T^ from closely related strains are shown in Table 1.

### Phylogenetic Analyses

**Fig. 1:**
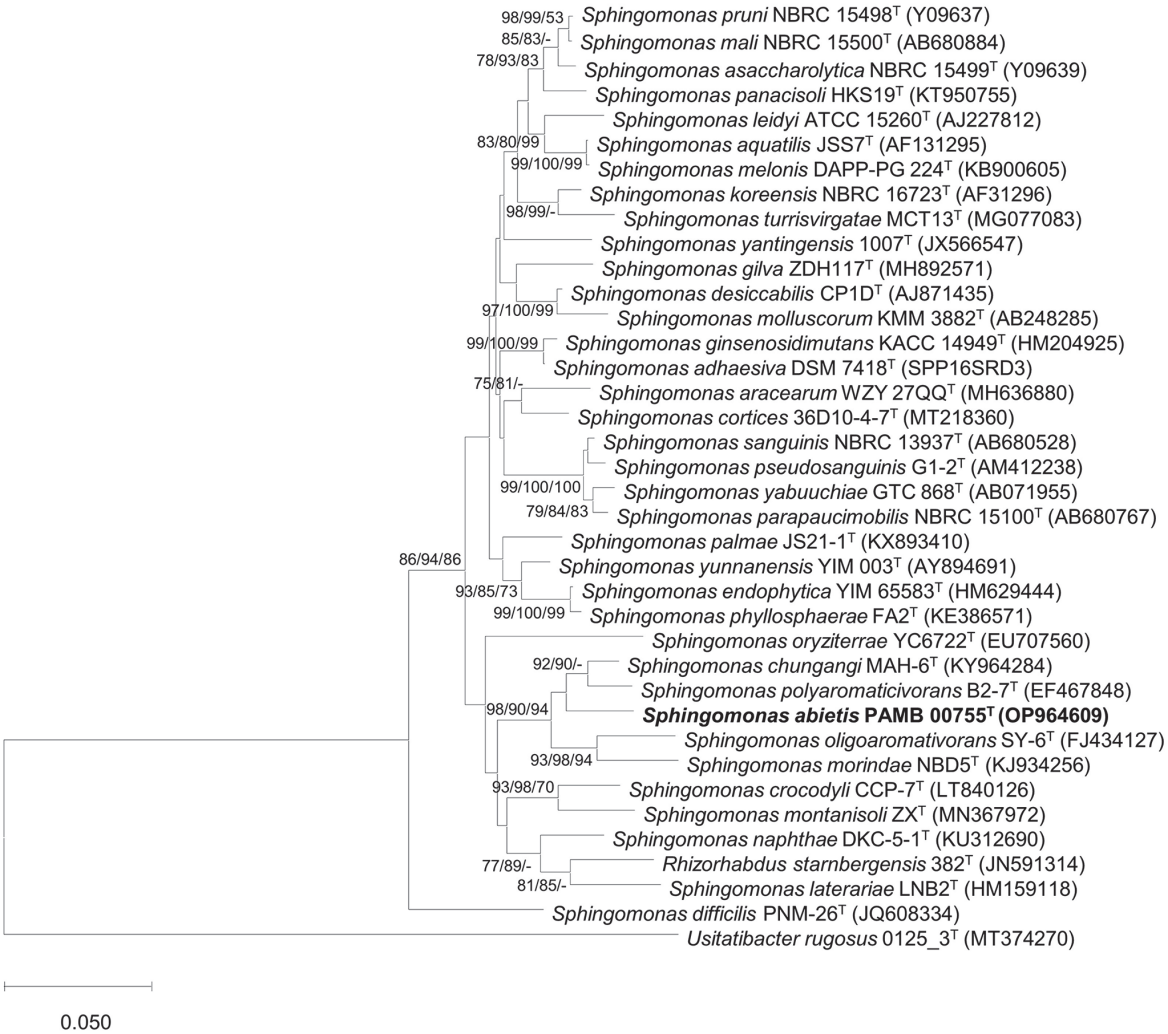
Phylogenetic analysis of the 16S rRNA gene in strain PAMB 00755^T^ and related species within the genus *Sphingomonas*. The presented values indicate bootstrap values (>70%) obtained using the neighbor-joining (NJ), maximum likelihood (ML), and minimum parsimony (MP) algorithms. The scale bar represents 0.050 substitutions per nucleotide position.

The complete 16S rRNA gene amplicon (1443 nucleotides) of strain PAMB 00755^T^ was obtained after assembly using Vector NTI software (1.6.1). Based on comparing the 16S rRNA sequence to the EzBioCloud and NCBI databases, strain PAMB 00755^T^ is related to members of the genus *Sphingomonas*, with the highest similarity to *S. chungangi* MAH-6^T^ (97.7%), *S. polyaromaticivorans* B2-7^T^ (97.4%), and *S. oligoaromativorans* SY-6^T^ (96.5%), and ≤96.0% similarity to other members of the genus *Sphingomonas*. In particular, the strain showed 93.0% 16S rRNA gene similarity to the recently proposed *Sphingomonas nostoxanthinifaciens* strain AK-PDB1-5^T^ based on using BioEdit software (7.2) [10]. Based on the novel species recognition cut-off value of < 98.6% [34], the strain was classified as a novel species of the genus *Sphingomonas*. To establish the phylogenetic position of the strain, a phylogenetic tree was constructed using the 16S rRNA gene sequence. The results showed that strain PAMB 00755^T^ clustered with *S. chungangi* MAH-6^T^ and *S. polyaromaticivorans* B2-7^T^ (Fig. 1). Based on the similarity of the 16S rRNA gene sequence and phylogenetic tree analysis, strain PAMB 00755^T^ was finally identified as a member of the *Sphingomonas*. To perform comparative analysis under identical conditions, *Sphingomonas*. *S. chungangi* MAH-6^T^ (= KACC 19292^T^) and *S. polyaromaticivorans* B2-7^T^ (= KCTC 82794^T^) were selected.

### Genomic and Phylogenomic Analysis

**Fig. 2:**
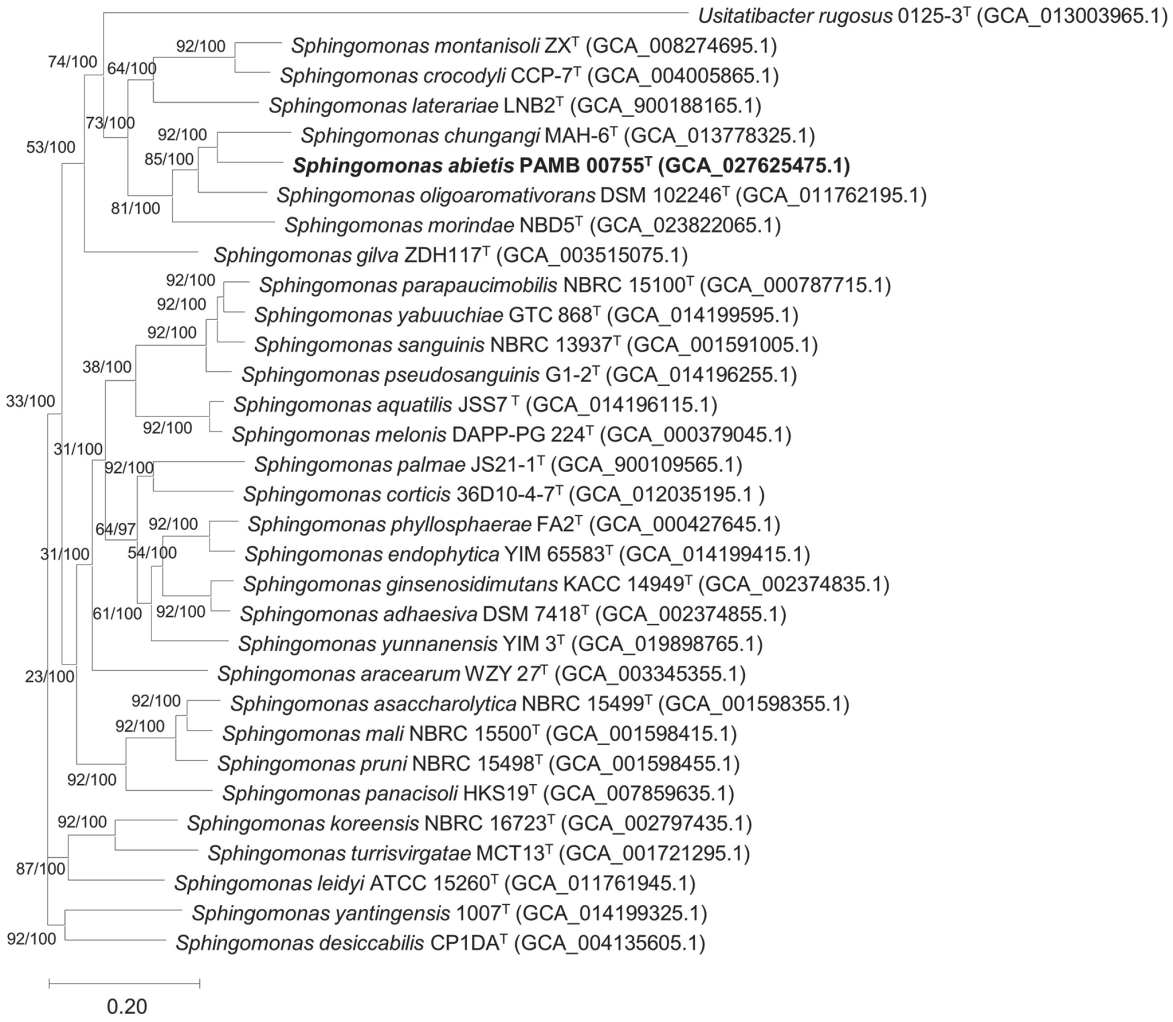
Phylogenomic tree of genus *Sphingomonas* based on the up-to-date bacterial core gene set (UBCG) showing the position of strain PAMB 00755^T^. At the nodes, the gene support index (GSI) is presented on the left side, while bootstrap values are indicated on the right side. The scale bar corresponds to 0.20 substitutions per position.

The complete genome of strain PAMB 00755^T^ was sequenced, resulting in a complete circular chromosome of 4,429,509 bp after *de novo* assembly using a microbial genome assembly application. The genome completeness, contamination, and quality values were determined to be 100, 0.9, and 95.5%, respectively. The G+C content of the genome was calculated to be 66.1%, similar to other species in the genus *Sphingomonas*, which have a high G+C content (57.4–70.5 mol%) (http://www.ncbi.nlm.gov/genome/?term=Sphingomonas). The genome annotation revealed the presence of 4,083 coding sequences (CDS), 59 tRNAs, and 12 rRNAs. Overall genome-related index (OGRI) analyses, involving dDDH, ANI, and AAI, were performed to determine the genetic relationships between strain PAMB 00755^T^ and closely related strains in the genus *Sphingomonas*. The dDDH values between strain PAMB 00755^T^ and its closely related strains of *S. chungangi* CGMCC 1.136^T^, *S. oligoaromativorans* DSM 102246^T^, *S. ginsenosidimutans* KACC 14949^T^, *S. adhaesiva* DSM 7418^T^, *S. endophytica* DSM 101535^T^, and *Sphingomonas morindae* NBD5^T^ were 24.3, 22.6, 19.7, 19.5, 19.7, and 20%, respectively. The ANI values between strain PAMB 00755^T^ and the abovementioned strains were 81.3, 79.9, 73.5, 73.4, 73.4, and 76.2%, respectively, while the AAI values were 75.9, 73.3, 59.2, 59.8, 59.5, and 67.1%, respectively. Considering the threshold values recommended for the bacterial species delineation, dDDH (<70%), ANI (<95–96%), and AAI (<95–96%) [35], strain PAMB 00755^T^ was identified as a novel strain in the genus *Sphingomonas*. The phylogenomic tree based on the whole genome also supported the placement of strain PAMB 00755^T^ within the genus *Sphingomonas* (Fig. 2). Fig. S2 shows a circular map of the strain PAMB 00755^T^ genome drawn using the CGView website. Annotation and analysis of secondary metabolite biosynthesis genes using anti-SMASH revealed that strain PAMB 00755^T^ contained a carotenoid biosynthesis cluster belonging to the terpenes (Table S1). The gene cluster involved in carotenoid biosynthesis could play an important role in antioxidant activity; however, further investigation is required to elucidate the exact bioactive chemicals and pathways for antioxidant activity.

### Chemotaxonomic Features

Table 2 shows that C_18:1_*ω*7*c* and/or C_18:1_*ω*6*c* (summed feature 8) (72.1%) is the major fatty acid of strain PAMB 00755^T^, which is similar to other closely related strains such as *S. chungangi* KACC 19292^T^ and *S. polyaromaticivorans* KCTC 82794^T^. However, *S. polyaromaticivorans* KCTC 82794^T^ had C19:0 cyclo ω8c (15.6%) as an additional major fatty acid, which distinguishes it from strain PAMB 00755^T^. The major polyamines in strain PAMB 00755^T^ were homospermidine (72.7%), putrescine (24.4%), and spermidine (2.4%) (Fig. S3), which is similar to other members of the genus *Sphingomonas* [16, 17, 20, 21]. The respiratory quinone of strain PAMB 00755^T^ was ubiquinone 10 (Q-10), consistent with other members of the genus *Sphingomonas*. The polar lipids in strain PAMB 00755^T^ were diphosphatidylglycerol (DPG), phosphatidylethanolamine (PE), sphingoglycolipid (SGL), phosphatidylcholine (PC), two unknown lipids (L1–L2), and four unknown aminolipids (AL1–AL4), phosphatidyl-N-methylethanolamine (PME), and an unknown aminophosphoglycolipid (APGL) (Fig. S4). Strain PAMB 00755^T^ had more polar lipid forms than other closely related strains.

Collectively, the finding from phylogenetic, genomic, phenotypic, and chemotaxonomic analyses indicate that strain PAMB 00755^T^ is a novel species within the genus *Sphingomonas*. The distinguishing phenotypic properties and chemotaxonomic data presented in Table 1, along with the low 16S rRNA gene sequence similarities (<97.7%), ANI (<81.3%), AAI (<75.9%), and dDDH (<24.3%) values compared to closely related strains in the genus *Sphingomonas*, support this conclusion. These values (16S rRNA similarities, ANI/AAI, dDDH) are much lower than the established thresholds for novel species recognition (98.6%, 95–96%, and 70%, respectively). The phylogenetic trees constructed using both the 16S rRNA and 92 core genes (according to the UBCG) derived from the whole-genome sequence also support this conclusion. In summary, strain PAMB 00755^T^ represents a new member of the genus *Sphingomonas*.

### Description of *Sphingomonas abietis*

***Sphingomonas abietis* (a.bi.étis. L. gen. n. *abietis*, of the silver fir, of the botanical genus *Abies*).** The strain forms circular, yellow colonies with a diameter of 2–4 mm when grown on PDA medium at 20°C for 3 days. It exhibits optimal growth on both PDA and R2A media at 20°C, pH 6.0, and 0–1% NaCl. The cells are Gram-negative, non-motile, short rods or ovoid (0.3–1.0 × 0.4–1.2 μm). The strain is catalase- and oxidase-positive. The strain exhibits positive reactions for various substrates as determined by API ZYM strips, including alkaline phosphate, esterase (C4), esterase lipase (C8), leucine arylamidase, valine arylamidase, cystine arylamidase, trypsin, a-chymotrypsin, acid phosphate, naphthol-AS-BI-phosphohydrolase, b-galactosidase, a-glucosidase, b-glucuronidase, and N-acetyl-b-glucosaminidase. Additionally, the API 20NE strips indicated positive reactions for esculine hydrolysis and b-galactosidase. As determined by API 50CH strips, it exhibits positive reactions for salicin, D-cellobiose, maltose, arbutin, esculin ferric citrate, D-lactose, and D-melezitose. The strain contains C_18:1_*ω*7*c* and/or C_18:1_*ω*6*c* (summed feature 8) as the major fatty acids and Q-10 as the respiratory quinone. Its major polar lipids include DPG, PE, SGL, PC, two unknown lipids (L1–L2), four unknown aminolipids (AL1–AL4), PME, and one unknown aminophosphoglycolipid (APGL).

Strain PAMB 00755^T^ is accessible from the Korea Collection for Type Culture (KCTC) with the designation KCTC 92781^T^ and the Guangdong Microbial Culture Collection Center (GDMCC) with the designation GDMCC 1.3779^T^. The accession numbers for its 16S rRNA and whole-genome sequences are OP964609.1 and CP115174.1, respectively. Based on its distinct phenotypic, genotypic, and chemotaxonomic characteristics, strain PAMB 00755^T^ was classified as a novel species within the genus *Sphingomonas*. Therefore, we propose the name *Sphingomonas abietis* sp. nov. for this newly identified species.

## Supplemental Materials

Supplementary data for this paper are available on-line only at http://jmb.or.kr.

## Figures and Tables

**Table 1:** Distinguishing physiological and biochemical characteristics of strain PAMB 00755^T^ and its closely related type strains.

Characteristic	1	2	3
Isolation source	Korea fir	Garden soil^a^	Oil port water^b^
Colony color	Yellow	Light yellow	Yellow^b^
Colony shape	Short rods or ovoid	Rods^a^	Short rods^b^
Oxidase	Positive	Weak^a^	Negative^b^
Colony size (mm)	2–4	0.6–1.2^a^	2–3^b^
Cell size (µm)	0.3–1.0 × 0.4–1.2	0.3–0.5 × 1.3–2.2^a^	1.04–1.90 × 0.52–0.75^b^
Growth at:			
Temperature (°C), range (optimum)	4–25 (20)	15–35 (30)^a^	20–35 (26)^b^
Optimum media	PDA and R2A	NA and R2A^a^	LB^b^
NaCl tolerance (%, w/v), range (optimum)	0–1 (0)	0–0.5 (0)^a^	0.5–3.0 (0.5)^b^
pH, range (optimum)	4.0–9.0 (6)	5.0–7.0 (6.5)^a^	4.0–7.5 (7)^b^
Assimilation (API 20NE) of:			
β-galactosidase	+	-	-
Glucose	-	-	+
Arabinose	-	-	+
API 50CH			
Arbutin	w	-	w
Esculin ferric citrate	w	-	w
Salicin	+	+	+
D-cellobiose	w	-	
Maltose	w	-	w
D-lactose	w	-	w
D-melezitose	w	-	w
API ZYM			
Trypsin	w	+	w
β-galactosidase	+	-	-
N-acetyl-β-glucosaminidase	+	-	+

Strains: 1, *Sphingomonas abietis* PAMB 00755^T^; 2, *S. chungangi* KACC 19292^T^; 3, *S. polyaromaticivorans* KCTC 82794^T^; “+”, positive, “w”, weakly positive, “−“, negative. Unless stated otherwise, all data are from our study. ^a^Akter and Huq, 2020, ^b^Luo, *et al*., 2012.

**Table 2:** Comparisons of cellular fatty acid profiles between strain PAMB 00755^T^ and closely related strains in the genus *Sphingomonas*.

	1	2	3
C_14:0_	ND	ND	0.8
C_16:0_	6.7	8.4	6.2
C_17:0_	1.1	ND	ND
C_18:0_	0.8	ND	0.5
C_17:1_*ω*6*c*	4.6	1.6	ND
C_18:1_*ω*5*c*	1.9	ND	1.7
C_19:0_ *cyclo ω8c*	ND	ND	**15.6**
C_12:0_-2OH	1.3	ND	0.7
C_13:0_-2OH	0.7	ND	ND
C_14:0_-2OH	6.8	8.5	7.3
C_15:0_-2OH	0.6	ND	ND
Summed feature 3^a^	1.1	ND	ND
Summed feature 8^a^	**72.1**	**81.5**	**67.2**

Strains: 1, *Sphingomonas abietis* PAMB 00755^T^; 2, *S. chungangi* KACC 19292^T^; 3, *S. polyaromaticivorans* KCTC 82794^T^; “ND” represents not detected. The major components (>10%) are highlighted in bold. All the data presented in this study were obtained from the present study. ^a^Summed features refer to groups of two or three fatty acids that cannot be individually distinguished using gas chromatography with the MIDI System. Summed feature 3 includes C_16:1_ *ω*6*c* and/or C_16:1_ *ω*7*c*, while summed feature 8 includes C_18:1_ *ω*7*c* and/or C_18:1_ *ω*6*c*.
