# Supplementary material for: Sphingomonas abietis sp. nov., an Endophytic Bacterium Isolated from Korean Fir
Source: J Microbiol Biotechnol. 2023 Jul 18;33(10):1292–8. doi: 10.4014/jmb.2303.03017 (PMC10619552; doi:10.4014/jmb.2303.03017)
Supplement: Supplementary file 1 [file jmb-33-10-1292-supple.pdf]

Supplementary Information

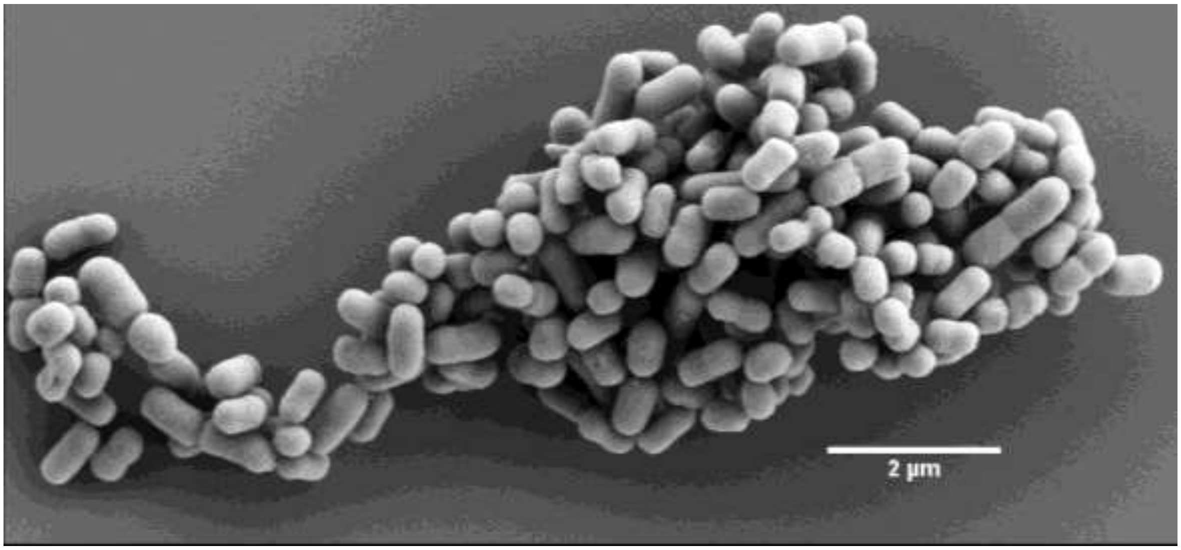

Fig. S1. Scanning electron micrograph of strain PAMB 00755^{T}. Scale bar = 2 μm.

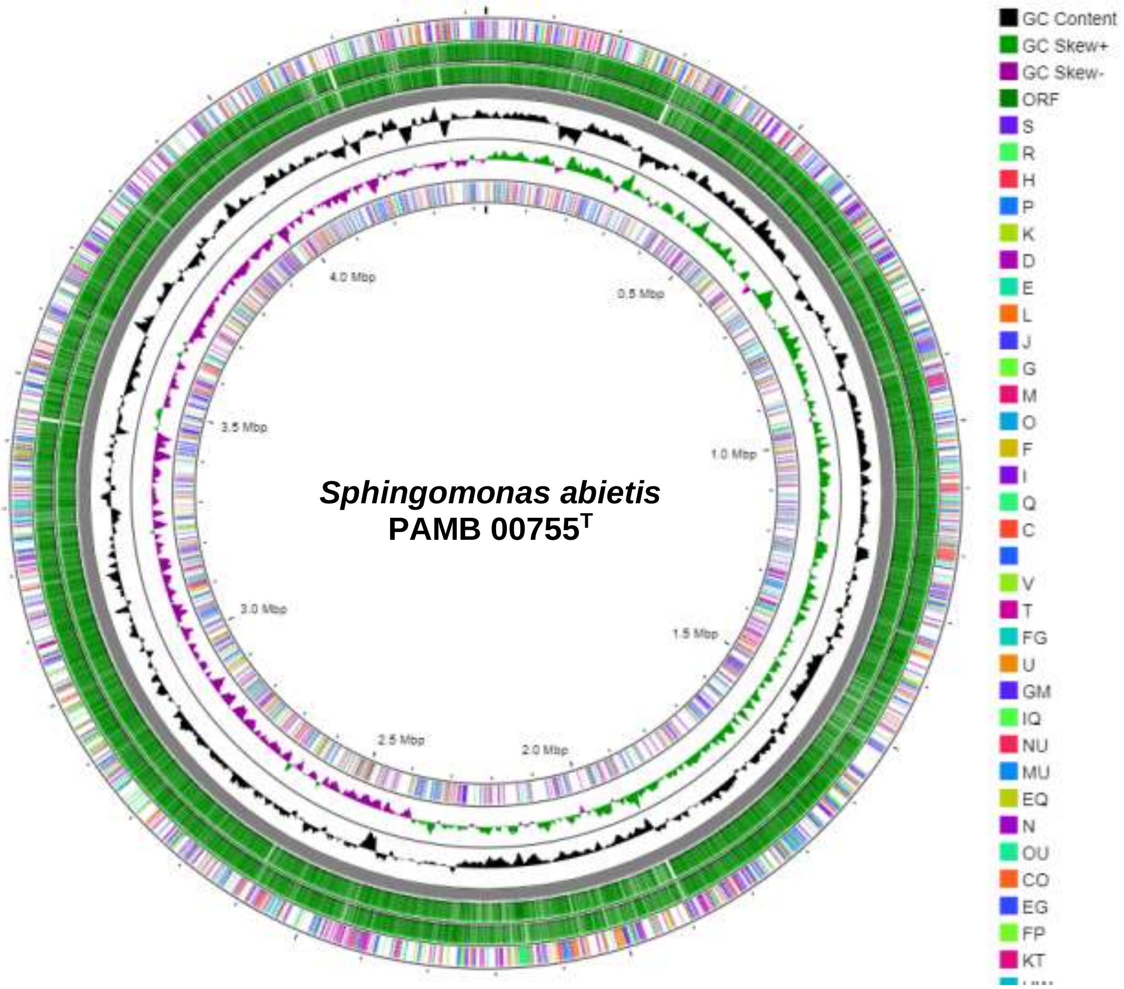

Fig. S2. Map of the *Sphingomonas abietis* PAMB 00755^{T} genome generated with CGView. Marked characteristics are shown from the outside to the center. Rings 1 and 6 show cluster orthologous group (COG) annotation in the forward and reverse directions, respectively. Ring 2 shows the GC skew, while ring 3 shows the G+C % content plot. Ring 4 and 5 showed the ORFs of the genome. The COG categories are: A, RNA processing and modification; B, chromatin structure and dynamics; C, energy production and conversion; D, cell cycle control, cell division, and chromosome partitioning; E, amino acid transport and metabolism; F, nucleotide transport and metabolism; G, carbohydrate transport and metabolism; H, coenzyme transport and metabolism; I, lipid transport and metabolism; J, translation, ribosomal structure, and biogenesis; K, transcription; L, replication, recombination, and repair; M, cell wall/membrane/envelope biogenesis; N, cell motility; O, post-translational modification, protein turnover, chaperones; P, inorganic ion transport and metabolism; Q, secondary metabolite biosynthesis, transport, and catabolism; R, general function prediction only; S, function unknown; T, signal

transduction mechanisms; U, intracellular trafficking, secretion, and vesicular transport; V, defense mechanisms; W, extracellular structures; X, mobilome: prophages, transposons; and Z, cytoskeleton.

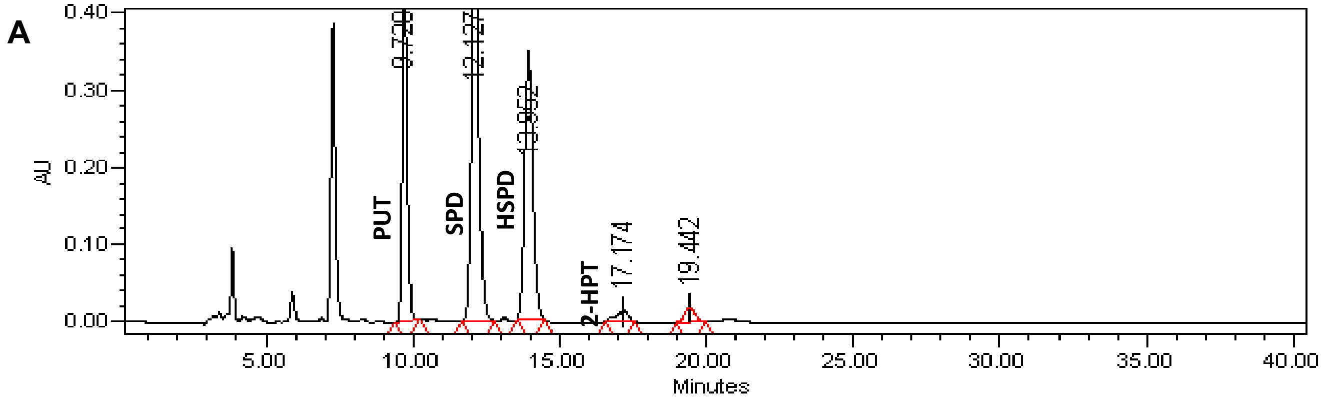

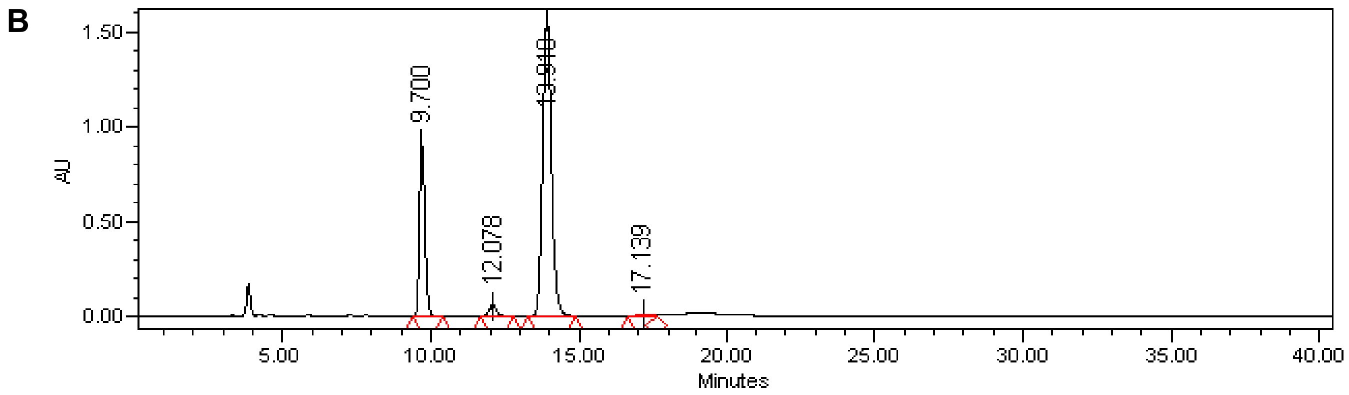

Fig. S3. HPLC chromatograms of polyamines. A) Polyamine standard. B) Strain PAMB 00755^{T}. PUT, putrescine (RT 9.720); SPD, spermidine (RT 12.127); HSPD, homospermidine (RT 13.952); 2-HPT, 2-hydroxyputrescine (RT 17.174).

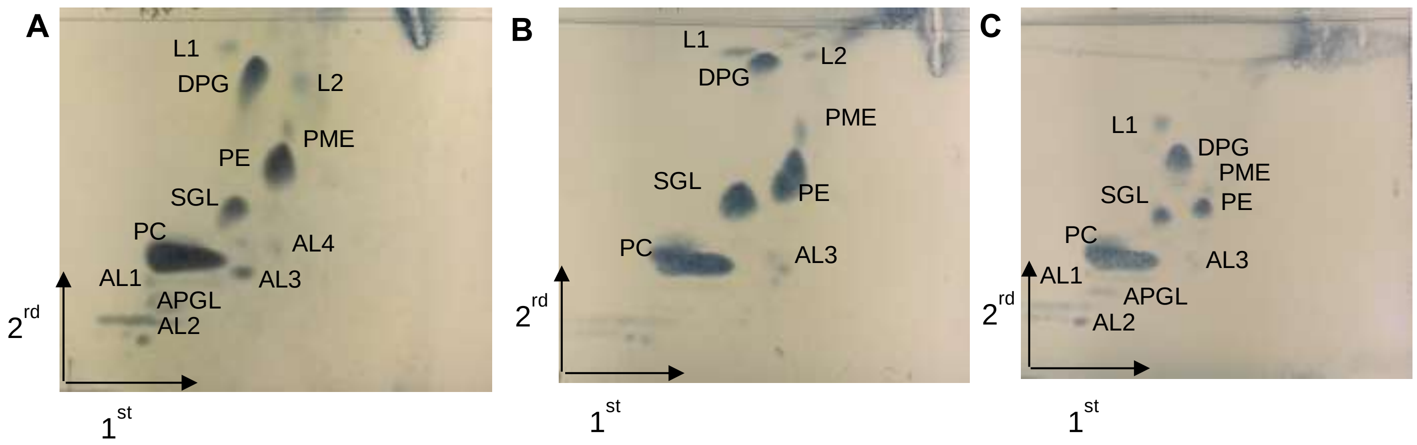

Fig. S4. Polar lipid profile of strain PAMB 00755^T and related type strains.

Strains: A) *Sphingomonas abietis* PAMB 00755^T; B) *S. chungangi* KACC 19292^T; C) *S. polyaromaticivorans* KCTC 82794^T. Diphosphatidylglycerol (DPG), phosphatidylethanolamine (PE), sphingoglycolipid (SGL), phosphatidylcholine (PC), unknown lipid (L1–L2), unknown aminolipids (AL1–4), phosphatidyl-N-methylethanolamine (PME), unknown aminophosphoglycolipid (APGL).

Table S1. Detection of secondary metabolite biosynthesis gene clusters in the genome sequence of strain AK-PDB1-5^{T} using antiSMASH.

Type	From	To	smBGC type	Most similar known cluster	Similarity	Core biosynthetic gene	Additional biosynthetic gene
Terpene	462,760	485,582				3	16
Lassopeptide	1,945,434	1,967,790	-	-	-	2	14
Lanthipeptide	3,036,081	3,077,918	-	-	-	2	29
Terpene	4,284,160	4,308,343	Carotenoid	Terpene	66%	2	4
